# Priapism After Initiation of Long-Acting Injectable Risperidone: A Case Report

**DOI:** 10.7759/cureus.108564

**Published:** 2026-05-09

**Authors:** António Santiago de Barros, Beatriz Freitas, Ana Catroga Nunes

**Affiliations:** 1 Psychiatry, Hospital Júlio de Matos-Unidade Local de Saúde de São José, Lisbon, PRT

**Keywords:** adverse drug reactions, antipsychotics, long-acting injectable antipsychotics, priapism, risperidone

## Abstract

Priapism is an uncommon but serious urological emergency, defined as a persistent penile erection unrelated to sexual stimulation. If not promptly treated, it carries a significant risk of permanent erectile dysfunction. Among pharmacologically induced causes, antipsychotic medications are frequently implicated. We describe the case of an 18-year-old male diagnosed with cannabis-induced psychosis who developed acute ischemic priapism within 24 hours of receiving his first dose of long-acting injectable risperidone. The patient required urgent urological management, which resulted in complete resolution of the episode. Risperidone was subsequently discontinued, and treatment was switched to olanzapine, gradually titrated to 10 mg/day. This change was associated with full remission of psychotic symptoms, without recurrence of priapism. This case underscores the importance of early recognition of priapism, careful antipsychotic selection, and patient counseling, particularly when initiating long-acting injectable formulations.

## Introduction

Priapism is an erectile disorder characterized by a persistent penile erection lasting longer than four hours and occurring independently of sexual stimulation. It is classified into ischemic (low-flow), nonischemic (high-flow), and recurrent ischemic subtypes, with ischemic priapism representing a urological emergency due to impaired venous outflow, tissue hypoxia, and risk of permanent erectile dysfunction if not promptly treated.

At a molecular level, penile erection is regulated by a complex interplay of neural, vascular, and biochemical pathways. Nitric oxide (NO) plays a central role by stimulating cyclic guanosine monophosphate (cGMP) production, leading to smooth muscle relaxation and increased cavernosal blood inflow. Detumescence, in turn, is mediated by sympathetic activation and degradation of cGMP. Priapism results from dysregulation of these pathways, particularly alterations in the NO-cGMP signaling cascade and impaired control of cavernosal smooth muscle tone.

Emerging evidence also highlights the involvement of additional molecular mediators, including adenosine signaling, Rho-kinase pathways, and phosphodiesterase type 5 (PDE5) activity, which together contribute to abnormal persistence of erection and defective detumescence [1].

Although uncommon, it represents a urological emergency, as delayed management may result in cavernosal ischemia, fibrosis of the corpora cavernosa, and permanent erectile dysfunction [2,3]. The ischemic (low-flow) subtype is the most frequent and clinically significant presentation.

Drug-induced priapism is a recognized etiology, with antipsychotic medications accounting for a considerable proportion of reported cases [4-6]. The proposed mechanism involves α1-adrenergic receptor antagonism in penile smooth muscle, leading to impaired sympathetic-mediated detumescence and prolonged cavernosal blood stasis [5,6]. Several second-generation antipsychotics with significant α1-adrenergic blockade, including risperidone, paliperidone, and clozapine, have been associated with priapism in case reports and pharmacovigilance data [4,7,8].

Among these, risperidone is one of the most frequently implicated agents, with multiple case reports and literature reviews supporting its association with priapism across different clinical contexts, including early treatment phases and dose adjustments [4]. The risk appears to correlate more closely with the degree of α1-adrenergic antagonism than with the antipsychotic class itself.

Despite its potential severity, priapism remains underrecognized in psychiatric practice. Increased clinical awareness is particularly important in young male patients and in those receiving long-acting injectable (LAI) antipsychotics, where rapid discontinuation is not immediately possible. We report a case of acute ischemic priapism occurring shortly after initiation of long-acting injectable risperidone and discuss its implications for psychiatric management.

## Case presentation

An 18-year-old male with no significant past medical history was admitted to the psychiatric inpatient unit due to an acute psychotic episode. The clinical presentation included paranoid delusions, behavioral disorganization, and marked agitation, occurring in the setting of recent cannabis use. A diagnosis of cannabis-induced psychosis was established. There was no personal or family history suggestive of hematologic conditions, urologic disorders, or prior episodes of priapism. No additional substance use was reported beyond cannabis, and there was no exposure to medications commonly linked to priapism. Cannabis use was considered a potential contributing factor; however, current evidence does not support a consistent direct association with priapism, and no use of other substances, such as cocaine or vasoactive agents, was reported.

Given the broad differential diagnosis of priapism, potential etiologies were systematically considered. Hematologic causes, particularly sickle cell disease, were considered less likely based on the absence of suggestive history and unremarkable laboratory findings, although they cannot be completely excluded. Drug-induced causes were reviewed, with no exposure to medications commonly associated with priapism other than risperidone, which had been recently initiated prior to symptom onset. There was no history of perineal trauma, malignancy, or use of vasoactive substances. No clinical or laboratory findings supported an alternative etiology. The absence of prior episodes and the temporal relationship between long-acting risperidone administration and symptom onset further supported a drug-induced mechanism.

Oral risperidone was initiated and progressively titrated to 9 mg daily to achieve symptom control after partial response at lower doses, resulting in significant symptomatic improvement and remission of psychotic features. Although most patients respond within the 2-8 mg/day range, higher doses may be required in some cases. This higher dose is reported to document overall exposure preceding the event, as antipsychotic-associated priapism is not clearly dose-dependent but rather related to pharmacodynamic mechanisms, including α1-adrenergic receptor blockade. Given concerns regarding long-term adherence, a transition to a long-acting injectable risperidone formulation was planned, and a 100 mg monthly intramuscular injection was administered, with no oral overlap required according to the formulation characteristics.

Within 24 hours of the first injection, the patient developed a sustained and painful penile erection lasting several hours. He was urgently referred to the emergency department, where the urology team confirmed the diagnosis of acute ischemic priapism. Bilateral corporal aspiration was performed, with drainage of stagnant cavernosal blood, achieving full detumescence and symptom resolution, without immediate complications.

After stabilization, the patient remained hospitalized for continued psychiatric monitoring. Risperidone was discontinued, and treatment with olanzapine was initiated and gradually titrated to 10 mg/day. The selection of olanzapine was supported by its comparatively lower α1-adrenergic receptor affinity and by previous reports describing successful treatment with olanzapine or aripiprazole following antipsychotic-associated priapism [8]. This strategy was intended to reduce, but not eliminate, the risk of recurrence, as rare cases of olanzapine-associated priapism have been reported. The patient was counseled regarding the potential risk of recurrence and advised to seek urgent medical evaluation if symptoms recur. The new regimen was well tolerated, with no recurrence of priapism or other significant adverse events. Under olanzapine therapy, the patient maintained full remission of psychotic symptoms and clinical stability. He was discharged on oral olanzapine 10 mg/day with outpatient psychiatric follow-up arranged. Written informed consent was obtained from the patient for publication of this case report.

## Discussion

This case illustrates a rare but clinically significant adverse reaction temporally associated with long-acting injectable risperidone. Symptom onset within 24 hours of administration supports a probable drug-related event, particularly in the absence of other identifiable predisposing factors. Acute ischemic priapism is a urological emergency requiring prompt evaluation and management, as highlighted in current American Urological Association/Sexual Medicine Society of North America (AUA/SMSNA) guidelines [9]. Based on the Naranjo adverse drug reaction probability scale [10], this case would be classified as a “probable” adverse drug reaction (Table 1).

**Table 1:** Naranjo Adverse Drug Reaction Probability Scale.

Question	Answer	Score
Are there previous conclusive reports on this reaction?	Yes	1
Did the adverse event appear after the drug was administered?	Yes	2
Did the adverse reaction improve when the drug was discontinued?	Yes	1
Are there alternative causes that could have caused the reaction?	No	2
Did the reaction reappear upon re-administration?	Not applicable	0
Was there a placebo response?	Not applicable	0
Was the drug detected in toxic concentrations?	Not applicable	0
Was the reaction more severe with increased dose or less with decreased dose?	Not applicable	0
Did the patient have a similar reaction to the same or similar drugs?	No	0
Was the adverse event confirmed by objective evidence?	Yes	1
Total score: 7-Probable adverse drug reaction		

Although alternative etiologies were considered, including substance-related causes, available evidence suggests that cannabis is only rarely associated with priapism, typically in recurrent forms, and data remain limited [11]. In the present case, the absence of prior episodes and the clear temporal relationship with risperidone administration make a substance-related etiology less likely.

Although antipsychotic-induced priapism has been described with multiple agents, risperidone is frequently reported, possibly due to its pharmacodynamic profile and widespread use [5,7]. Importantly, available evidence suggests that this adverse effect is unpredictable and not clearly dose-dependent. Episodes have been documented early in treatment, after dose adjustments, and even during stable maintenance therapy [4,7], indicating that individual susceptibility may play a relevant role.

Long-acting injectable formulations introduce additional clinical complexity, as drug exposure cannot be rapidly withdrawn once administered. Reports of priapism following conversion from oral to injectable risperidone or paliperidone highlight the need for careful monitoring during the initial post-injection period [7,8]. This is particularly relevant in young male patients, in whom delayed recognition may have long-term functional consequences.

Following resolution of the acute episode, reassessment of antipsychotic therapy is essential. Although no formal guidelines dictate subsequent pharmacological strategy, switching to an agent with comparatively lower α1-adrenergic receptor affinity has been suggested in previous reports [4,6,8]. In the present case, transition to olanzapine resulted in sustained psychiatric stability without recurrence of priapism, supporting this approach.

Finally, this case underscores the importance of proactive patient education. Individuals initiating antipsychotic treatment, especially long-acting formulations, should be informed about early warning signs and instructed to seek urgent medical evaluation if symptoms arise. Prompt intervention remains critical to preventing irreversible complications and maintaining adherence to psychiatric care [2].

## Conclusions

This case highlights an uncommon but clinically relevant complication temporally associated with long-acting injectable risperidone. The event underscores the importance of close monitoring during treatment transitions and timely urological evaluation when suggestive symptoms arise. Reassessment of antipsychotic therapy after such episodes is essential, and alternative agents may be safely implemented. Clinician awareness and patient counseling remain key measures to reduce morbidity and support continuity of psychiatric care.
